# Plant-Derived Modulators of Tumor Metabolism as Novel, Efficacious, and Low-Toxicity Therapeutic Agents for Cancer Treatment

**DOI:** 10.3390/molecules31091394

**Published:** 2026-04-23

**Authors:** Tania Mmapule Maphoso, Dakalo Portia Ramali, Thanyani Mulaudzi, Vinesh Maharaj, Cathryn Helena Stanford Driver, Botle Precious Damane

**Affiliations:** 1 Department of Surgery, Level 7, Bridge E, Steve Biko Academic Hospital, Faculty of Health Sciences, University of Pretoria, Pretoria 0001, South Africa; tania.maphoso@up.ac.za (T.M.M.); u18068449@tuks.co.za (D.P.R.); thanyani.mulaudzi@up.ac.za (T.M.); 2 Biodiscovery Centre, Department of Chemistry, Faculty of Natural and Agricultural Sciences, University of Pretoria, Pretoria 0028, South Africa; vinesh.maharaj@up.ac.za; 3 Nuclear Medicine Research Infrastructure NPO, Level 4, Steve Biko Academic Hospital, Pretoria 0001, South Africa; cathryn.driver@sanumeri.co.za; 4 Department of Nuclear Medicine, Level 5, Steve Biko Academic Hospital, Faculty of Health Sciences, University of Pretoria, Pretoria 0001, South Africa; 5 South African Nuclear Energy Corporation, Elias Motsoaledi St., R104 Pelindaba, Brits Municipality, Pretoria 0240, South Africa

**Keywords:** phytochemicals, cancer metabolism, tumor microenvironment, metabolic reprogramming, metabolic modulators

## Abstract

Metabolic reprogramming is a core hallmark of malignancy, enabling tumor cells to sustain rapid proliferation, evade immune elimination, and develop resistance to therapy. Although a wide range of plant-derived phytochemicals exhibit anticancer activity with comparatively low toxicity, their capacity to disrupt specific metabolic dependencies exploited by tumors has not been comprehensively synthesized. This review brings together current mechanistic evidence showing how major phytochemical classes, including polyphenols, terpenes and terpenoids, glucosinolates, and alkaloids, interfere with pathways central to tumor metabolic fitness, such as aerobic glycolysis, pentose phosphate pathway flux, mitochondrial substrate oxidation, glutamine dependence, and redox homeostasis. It further introduces a pathway-focused framework that links phytochemical mechanisms to quantifiable metabolic outcomes and highlights their potential to remodel the tumor microenvironment by altering nutrient competition, oxidative stress responses, and hypoxia-driven signaling. Key barriers such as poor systemic bioavailability, rapid metabolic degradation, and limited tissue penetration are assessed alongside emerging formulation and delivery strategies designed to enhance therapeutic exposure while preserving low-toxicity profiles. Mapping these mechanistic insights onto clinical development needs allows prioritization of specific phytochemical-metabolic pathway pairs with the strongest potential for translation. This positions plant-derived metabolic disruptors as promising candidates for next-generation, low-toxicity anticancer therapies that strategically exploit defined metabolic vulnerabilities.

## 1. Introduction

Despite established clinical guidelines, standard cancer treatments often cause serious adverse effects and are limited in their long-term tolerability. Chemotherapy, for example, can induce multi-organ toxicities, including nephrotoxicity, hepatotoxicity, neurotoxicity, cardiotoxicity, and myelosuppression [1]. In addition, treatment resistance frequently emerges from altered drug metabolism, enhanced efflux of chemotherapeutic agents, and changes in cellular signaling pathways [2]. Targeted and immune-mediated therapies represent promising advances, but adverse effects, treatment resistance, and high costs continue to limit their impact on mortality and survival in metastatic cancer [3]. Radiopharmaceuticals offer a novel diagnostic and therapeutic option for solid tumors and metastases, although reliance on multiple scans may increase healthcare costs and logistical burden [4,5]. Together, these factors underscore the importance of exploring novel therapeutic approaches. Metabolic plasticity within the tumor microenvironment (TME) has emerged as a central therapeutic vulnerability in cancer, a concept rooted in Otto Warburg’s early observation that cancer cells preferentially rely on aerobic glycolysis, converting glucose to lactate despite sufficient oxygen. This altered metabolic pattern is known as the Warburg effect [6,7]. This foundational insight into cancer metabolism has inspired the development of therapies targeting metabolic vulnerabilities, including mutant isocitrate dehydrogenase 1 and 2 (IDH1/2) inhibitors, glutaminase (GLS) inhibitors, and fatty acid synthase inhibitors [8,9,10]. Although some, such as ivosidenib and enasidenib, are approved for acute myeloid leukemia, most agents remain in preclinical or early clinical trials, highlighting the limited availability of established metabolic-targeted therapies [11].

This is where phytochemicals may offer a clinical advantage as a novel therapeutic approach. These naturally occurring compounds are potential alternatives or complements to conventional cancer therapies, exhibiting diverse bioactivities that can target tumor growth and survival [12]. Notable for their ability to modulate key signaling pathways, act as chemopreventive agents, and enhance the efficacy of immunotoxins, phytochemicals may also influence the metabolic adaptations that enable cancer cell survival, providing a unique avenue to exploit tumor-specific vulnerabilities [13]. With the TME playing a major role in therapeutic responses and resistance, phytochemicals have been shown to modulate the TME by restoring immune cell function, including reactivating T cells, reducing pro-inflammatory signaling, and promoting the polarization of macrophages from a pro-tumor (M2-like) to an anti-tumor (M1-like) phenotype [14]. Chiou et al. [15] showed that piceatannol (PIC) inhibits M2 polarization of tumor-associated macrophages (TAMs) in colon cancer, reducing transforming growth factor-beta 1 (*TGF-β1*), arginase 1 (*Arg1*), CD163, and fibronectin. This reprogramming suppressed TAM-driven migration, invasion, epithelial–mesenchymal transition, and stemness in SW480 cells. In vivo, PIC limited tumor growth and metastasis, partly by directly targeting TGF-β receptor 1 (*TGF-βR1*), highlighting its potential to modulate the TME and curb colorectal cancer progression [15].

Thus, phytochemical-mediated disruption of energy and biosynthetic pathways may play a critical role in reducing therapy resistance by limiting drug efflux, DNA repair capacity, and other metabolic survival mechanisms. These compounds may also modulate the TME by altering nutrient availability, redox balance, and hypoxia, collectively creating conditions less favorable for tumor growth [16]. By emphasizing the systemic and interconnected nature of tumor metabolism, this review provides a framework to understand how multiple metabolic pathways and immunometabolic interactions can be targeted simultaneously, offering insights into combinatorial strategies and mechanistic links that support therapeutic efficacy. Building on these insights, this review examines the key metabolic adaptations that enable cancer cell survival and proliferation, providing mechanistic insights into how plant-derived phytochemicals can selectively disrupt these pathways. Where available, we highlight studies that report quantifiable metabolic outcomes, linking specific compounds to measurable changes in glycolysis, pentose phosphate pathway flux, mitochondrial activity, and redox balance. We further emphasize how phytochemicals modulate the TME, including immune cell metabolism and polarization, nutrient competition, and hypoxia-driven signaling, offering a comprehensive perspective on TME immunometabolism. By evaluating evidence from both experimental and clinical studies, this review provides a framework to prioritize phytochemical-metabolic pathway pairs with the strongest translational potential.

## 2. Methodology

A comprehensive literature search was conducted to identify peer-reviewed studies examining cancer metabolism and the role of plant-derived phytochemicals in modulating metabolic pathways associated with tumor progression. Electronic databases, including Google Scholar (https://scholar.google.com (accessed on 1 September 2025)), PubMed (https://pubmed.ncbi.nlm.nih.gov (accessed on 1 September 2025)), Web of Science (https://www.webofscience.com (accessed on 1 September 2025)), and Scopus (https://www.scopus.com (accessed on 1 September 2025)), were systematically searched. The search strategy incorporated combinations of keywords such as cancer metabolism, metabolic reprogramming, Warburg effect, TME, phytochemicals, polyphenols, terpenoids, alkaloids, and related synonyms. The search was primarily restricted to studies published between 2015 and 2025 to ensure scientific relevance and currency. Earlier landmark publications were selectively included where necessary to provide foundational insights into key metabolic mechanisms. Eligible articles were required to be peer-reviewed, published in English, and to directly investigate the interaction between phytochemicals and metabolic pathways involved in cancer development or progression. Both experimental and review studies that provided mechanistic insights into metabolic modulation were considered. Studies that did not focus on cancer metabolism, lacked mechanistic relevance, or were not peer-reviewed were excluded from the analysis. This work follows a narrative review approach, where studies were selected and synthesized based on their scientific relevance, methodological quality, and contribution to understanding how phytochemicals influence metabolic reprogramming in cancer cells and the TME.

## 3. Pathways Involved in Metabolic Reprogramming in Cancer Cells

The architecture of metabolic reprogramming spans multiple levels, from genomic and proteomic alterations to cellular and tissue-level adaptations. These changes are orchestrated to maximize energy production and provide the biosynthetic building blocks necessary for rapid tumor growth. Oncogenic drivers remodel key signaling pathways, producing metabolic outputs that support tumor survival and proliferation. This reprogramming confers metabolic plasticity, enabling cancer cells to thrive under fluctuating oxygen levels and nutrient [17]. Given the centrality of these processes to tumor progression, they represent strategic therapeutic vulnerabilities. The key metabolic pathways that sustain tumor growth and offer opportunities for targeted intervention are explored in the following subsections.

### 3.1. Aerobic Glycolysis and the Warburg Effect

**Figure 1:**
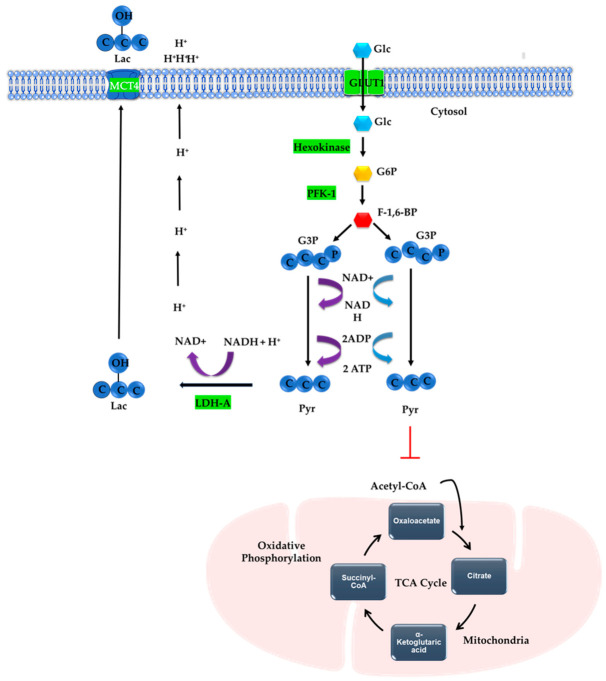
Altered glucose metabolism in cancer cells. Glucose (Glc) enters the cancer cell through glucose transporter 1 (GLUT1) and is converted into glucose-6-phosphate (G6P), trapping it inside the cell. G6P is then transformed into fructose-1,6-bisphosphate (F-1,6-BP), which is further broken down into glyceraldehyde-3-phosphate (G3P). Subsequent reactions convert G3P into pyruvate (Pyr), utilizing nicotinamide adenine dinucleotide (NAD^+^) and producing reduced nicotinamide adenine dinucleotide (NADH), while simultaneously generating adenosine triphosphate (ATP) from adenosine diphosphate (ADP). Instead of entering the tricarboxylic acid (TCA) cycle and undergoing oxidative phosphorylation, pyruvate is preferentially converted into lactate (Lac) by lactate dehydrogenase A (LDH-A). This reaction regenerates NAD^+^ by oxidizing NADH, ensuring continuous glycolytic flux. The accumulated lactate, along with protons (H^+^), is exported out of the cell via monocarboxylate transporter 4 (MCT4). This process contributes to extracellular acidification, promoting a tumor-supportive microenvironment by enhancing immune evasion, angiogenesis, and metastasis.

In healthy cells, glycolysis is a 10-step process that generates a net gain of 2 adenosine triphosphate (ATP) molecules and produces 2 pyruvate molecules. In the presence of oxygen, these pyruvate molecules enter oxidative phosphorylation, yielding approximately 30–36 ATP molecules in total [18]. However, in cancer cells, pyruvate is not efficiently funneled into oxidative phosphorylation but is instead reduced to lactate (Figure 1), enabling the regeneration of nicotinamide adenine dinucleotide (NAD^+^) [17]. Although the mitochondria remain functional and a portion of pyruvate continues to fuel oxidative phosphorylation, emerging interpretations propose that high glycolytic flux may exceed mitochondrial metabolic capacity, a phenomenon described as mitochondrial overload. This limitation constrains oxidative metabolism and favors the diversion of pyruvate toward lactate, enabling cancer cells to maintain redox balance and limit excessive reactive oxygen species (ROS) [19].

Beyond its role in energy production, lactate accumulation drives proton efflux and acid export into the extracellular matrix, contributing to TME acidification and promoting tumor progression. This metabolic phenotype is reinforced by oncogenic signaling and genetic alterations that enhance glycolytic enzyme activity and regulatory control [20]. A key mechanism sustaining high glycolytic flux is the upregulation of glucose and lactate transporters in cancer cells [21], which have gained prominence as theragnostic targets in radiolabeling-based diagnostic and therapeutic strategies [22]. Among these, glucose transporter 1 (GLUT1) and glucose transporter 3 (GLUT3) are the primary glucose importers in tumors, with their expression strongly induced by hypoxia in the TME [23]. Hypoxia not only upregulates GLUT1 and GLUT3 to increase glucose uptake but also induces monocarboxylate transporter 4 (MCT4), which facilitates lactate export. Together, these transporters maintain elevated glycolytic flux, sustain extracellular acidification, and promote immune evasion. Lactate dehydrogenase (LDH) further drives this pathway by converting pyruvate to lactate, as illustrated in Figure 1 [24]. This metabolic architecture is regulated by key rate-limiting enzymes, including hexokinase (HK), phosphofructokinase 1 (PFK-1), and pyruvate kinase M2 (PKM2) [25]. In many cancers, HK, PFK-1, and LDH are further upregulated by pathways such as the cellular Myelocytomatosis oncogene (c-Myc) oncogene, hypoxia-inducible factor 1 alpha (HIF-1α), and the PI3K/Akt/mTOR signaling pathway [25]. These metabolic alterations secure a constant supply of ATP and maintain an acidic environment that favors cancer progression.

### 3.2. The Pentose Phosphate Pathway

Cancer cells exploit alternative metabolic pathways such as the pentose phosphate pathway (PPP). The PPP is a critical glycolytic shunt that supplies both reducing power and ribonucleotides, enabling cancer cells to sustain proliferation and survive oxidative stress. This pathway consists of two distinct branches: the oxidative arm, which generates nicotinamide adenine dinucleotide phosphate (NADPH), and the non-oxidative arm, which supplies ribose-5-phosphate for nucleotide synthesis [26]. G6PD catalyzes the rate-limiting step of the oxidative phase of the PPP by oxidizing (G6P) to 6-phosphogluconolactone (6-PGL), which is subsequently converted into 6-phosphogluconate (6-PG) [27]. Cancer cells channel G6P into the PPP to simultaneously generate NADPH for oxidative stress resistance and ribose-5-phosphate for nucleotide synthesis. This metabolic shift is driven by increased G6PD activity and loss of TP53 regulation, enabling tumor cells to maintain redox balance while sustaining rapid proliferation [28]. Functionally, G6PD overexpression provides a selective advantage to tumor cells by strengthening antioxidant defenses and increasing biosynthetic output. However, this same pro-tumor activity translates clinically into worse patient outcomes. A pan-cancer analysis by Liu et al. [29] found G6PD to be highly expressed in hepatocellular carcinoma, glioma, and breast cancer, with elevated levels correlating with poor prognosis [29]. Several proteins positively regulate G6PD and the PPP. HIF-1α and nuclear factor erythroid 2-related factor 2 (Nrf2) enhance PPP flux under hypoxic or oxidative stress conditions. Kinases such as ataxia telangiectasia mutated (ATM) and protein kinase B (AKT/PKB) stimulate G6PD activity through post-translational modifications. In contrast, tumor suppressors TP53 and AMP-activated protein kinase (AMPK) act as negative regulators by limiting G6P entry into the PPP, thereby restricting nucleotide synthesis and antioxidant capacity [28,29]. Loss of TP53, a common event in tumors, leads to upregulation of the PPP and enhances survival under oxidative stress. This regulatory link is exemplified by Tang et al. [30], who showed that the oncogene p52-ZER6 promotes tumorigenesis by facilitating TP53 ubiquitination and degradation while simultaneously enhancing aerobic glycolysis. Their study further demonstrated that p52-ZER6 directly upregulates G6PD transcription, activating the PPP and increasing nucleotide and NADPH production to support tumor and survival [30]. Similarly, p21-activated kinase 4 inhibits TP53, contributing to dysregulated glucose-6-phosphate metabolism. Although PAK4 is dysregulated in several cancer types, its metabolic role in colon cancer has been shown to enhance glucose uptake, NADPH production, and lipid biosynthesis to support proliferation. Mechanistically, PAK4 interacts with G6PD and promotes Mdm2-mediated TP53 degradation [31]. Other enzymes within the PPP that are dysregulated in cancer include 6-phosphogluconate dehydrogenase (6PGD), transketolase (TKT), transketolase-like 1 (TKTL1), and ribose-5-phosphate isomerase (RPIA). 6PGD drives the oxidative decarboxylation of 6-phosphogluconate, contributing to NADPH production [28]. TKT supports carbon rearrangement in the non-oxidative branch of the PPP. Its homolog, TKTL1, is frequently overexpressed in tumors and further amplifies non-oxidative PPP flux. RPIA converts ribulose-5-phosphate into ribose-5-phosphate, providing the ribose backbone required for nucleotide synthesis. This upregulation of the PPP increases NADPH and ribose-5-phosphate availability, enhancing antioxidant defenses and nucleotide synthesis, thereby enabling cancer cells to proliferate and survive oxidative stress [28]. These alterations in the PPP increase metabolic adaptability, highlighting the pathway’s central role in supporting cancer cell survival [21].

### 3.3. Lipid Metabolism and Membrane Biosynthesis

**Figure 2:**
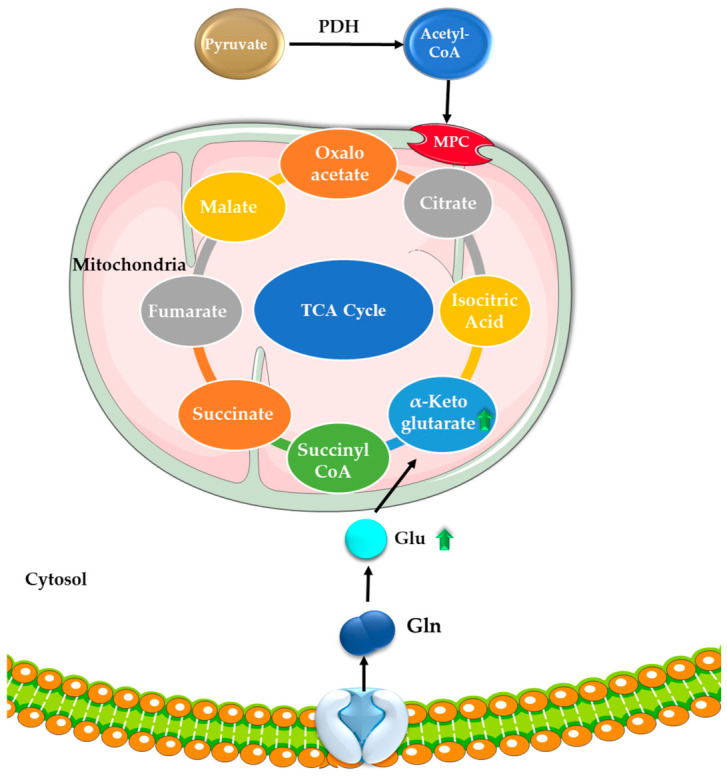
Altered tricarboxylic acid (TCA) cycle and glutamine addiction. Pyruvate generated from glycolysis is converted by pyruvate dehydrogenase (PDH) into acetyl-CoA, linking glycolysis to the tricarboxylic acid (TCA) cycle. Pyruvate enters the mitochondria using the mitochondrial pyruvate carrier (MPC), where it fuels this process. Cancer cells often rely on glutamine (Gln), which is converted to glutamate (Glu) and subsequently to α-ketoglutarate (α-KG), replenishing TCA cycle intermediates and supporting biosynthesis. Green arrows indicate upregulated metabolites in cancer cells, sustaining rapid proliferation through enhanced energy production and macromolecule synthesis.

In healthy cells, the tricarboxylic acid (TCA) cycle plays a crucial role in energy production, biosynthesis, and redox balance by oxidizing acetyl-CoA into carbon dioxide [32]. However, in cancer cells, the TCA cycle is frequently rewired to support rapid proliferation, with alterations in key enzymes such as succinate dehydrogenase (SDH), α-ketoglutarate dehydrogenase (α-KGDH), and fumarate hydratase (FH) [33]. One factor contributing to this metabolic reprogramming is the increased export of citrate, synthesized from acetyl-CoA and oxaloacetate and redirected toward fatty acid synthesis [34]. Cancer cells also display an increased dependence on lipids and cholesterol, supported by either enhanced uptake of exogenous lipids and lipoproteins or upregulated endogenous lipid synthesis. This metabolic shift often results in excessive lipid droplet accumulation, which is now recognized as a marker of cancer aggressiveness [35]. In addition, pyruvate generated from glycolysis contributes to lipid synthesis through its conversion to acetyl-CoA by pyruvate dehydrogenase (PDH) (Figure 2). This increased acetyl-CoA availability enhances fatty acid synthesis and supports uncontrolled cancer cell growth by facilitating membrane biogenesis and energy storage [36].

Several enzymes and regulatory proteins coordinate the enhanced fatty acid metabolism observed in cancer cells. Stearoyl-CoA desaturase-1 (SCD1) is frequently overexpressed in cancer and promotes cell proliferation and survival by converting saturated fatty acids into monounsaturated fatty acids, thereby reducing lipid-induced endoplasmic reticulum stress. This is demonstrated by Luis et al. [37], who examined the TME and found that SCD1, together with fatty acid-binding protein 4 (FABP4), are essential for tumor relapse following tyrosine kinase inhibitor or chemotherapy treatment. In the TME, FABP4 facilitates lipid transfer, supporting tumor recurrence, a finding confirmed in their study by analyzing its expression and functional impact on cancer cell survival [37]. Upstream, signal transducer and activator of transcription 3 (STAT3), orchestrates fatty acid synthesis by activating SCAP and sterol regulatory element-binding proteins (SREBF1) which in turn increases SCD1 expression and overall lipogenesis. Fan et al. [38] validated this mechanism by showing co-expression of STAT3, SCAP, SREBP-1, and SCD1 in glioblastoma samples, linking this regulatory axis to tumor progression [38]. These coordinated mechanisms drive lipid production, membrane biogenesis, and energy storage, sustaining tumor growth and highlighting potential metabolic vulnerabilities.

### 3.4. Glutamine Addiction and One-Carbon Metabolism

In healthy cells, glutamine supports energy production, biosynthesis, and one-carbon metabolism, providing essential units for nucleotide synthesis and epigenetic regulation. In cancer cells, this system is hijacked: increased glutamine uptake fuels rapid energy production and anabolic growth while supplying carbon and nitrogen for nucleotide and amino acid biosynthesis, as well as one-carbon units that drive uncontrolled proliferation and epigenetic remodeling [39]. Glutamine uptake is primarily mediated by the solute carrier family 1-member A5 (SLC1A5), which facilitates its transport into the cell, while the solute carrier family 7-member A5 (SLC7A5) functions as an antiporter, exporting intracellular glutamine in exchange for essential amino acids such as leucine. This coordinated transport mechanism supports intracellular amino acid homeostasis and drives activation of growth-promoting signaling pathways, including The Mammalian/Mechanistic Target of Rapamycin Complex 1 (mTORC1). In cancer, these transporters are frequently overexpressed, particularly in the context of *MYC* and *KRAS* mutations, thereby enhancing metabolic reprogramming and sustaining proliferative signaling. Once converted to glutamate by GLS, glutamine feeds into the TCA cycle as α-KGDH, replenishing intermediates and sustaining diverse biosynthetic processes [39,40,41]. This conversion occurs through aminotransferases such as glutamate dehydrogenase 1 (GLUD1), aspartate aminotransferase 2 (GOT2), and alanine aminotransferase 2 (GPT2), generating aspartate and alanine in the process. These amino acids are essential for nucleotide and protein synthesis. Glutamine metabolism also contributes to NADPH production, maintaining redox balance in cancer cells [39]. Glutamate derived from glutamine feeds indirectly into serine biosynthesis, linking glycolysis, glutaminolysis, and one-carbon metabolism [42]. Serine, in turn, enters the folate cycle, methionine cycle, and trans-sulfuration pathway, which collectively support nucleotide synthesis and methylation reactions [43]. These interconnected pathways generate S-adenosylmethionine (SAM), the universal methyl donor required for DNA and histone methylation, thereby promoting epigenetic changes that drive cancer progression [44]. Targeting glutamine metabolism through GLS inhibitors or disrupting one-carbon metabolism represents a promising strategy to selectively impair cancer cell survival while sparing normal tissues.

### 3.5. Metabolic Reprogramming in the Tumor Microenvironment

**Figure 3:**
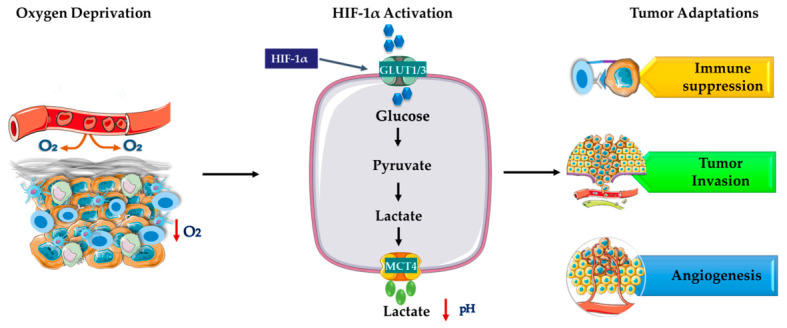
Metabolic reprogramming of tumor cells under hypoxic conditions and the consequences within the tumor microenvironment. With oxygen failing to penetrate the tumor microenvironment, HIF-1α is stabilized, leading to the upregulation of glucose transporter 1 (GLUT1) and glucose transporter 3 (GLUT3), which enhance glucose uptake. Increased glycolysis results in the conversion of glucose into pyruvate, which is further metabolized into lactate. Lactate is exported out of the cell via monocarboxylate transporter 4 (MCT4), leading to an accumulation of lactate in the extracellular space. This results in a decrease in pH, creating an acidic TME that suppresses immune responses by inhibiting cytotoxic T-cell function and reducing antigen presentation, contributing to immune evasion. Additionally, the low pH promotes tumor invasion by altering the extracellular matrix and enhancing cancer cell motility. Hypoxia and lactate also upregulate vascular endothelial growth factor (VEGF), stimulating angiogenesis, which facilitates tumor growth and progression.

Metabolic reprogramming in the TME also leads to immune evasion by modulating immune cell metabolism and function (Figure 3) [45]. Glucose levels in the TME are critical for immune cell function, as glucose is the primary energy source for immune cells, particularly T cells, supporting the metabolic pathways necessary for their activation. Glucose also fuels macrophages, supporting their roles in cytokine production, antigen presentation, and phagocytosis [46]. Regulatory T cells also show increased glucose uptake to sustain their immunosuppressive activity, generating key metabolites necessary for survival in the TME [47]. Cancer cells overexpress glucose transporters to ensure a constant glucose supply for their aggressive growth [48]. The competition for glucose in the TME illustrates the critical role of glucose transporters in regulating both immune and cancer cell functions [47]. In addition, HIF-1α stabilization enhances glycolysis and lactate production, leading to an acidified extracellular space that suppresses immune function [49]. TAMs polarize toward an immunosuppressive phenotype, while cytotoxic T-cell activity declines due to increased programmed death-ligand 1 (PD-L1) expression [50]. Hypoxia also suppresses dendritic cell maturation, promotes neutrophil extracellular trap formation, and stimulates cancer-associated fibroblasts to release vascular endothelial growth factor (VEGF) and interleukin-6 (IL-6), reinforcing a tumor-supportive microenvironment [51]. Overall, the metabolic rewiring induced by hypoxia, nutrient stress, and glycolytic acidosis coordinates immunosuppression, angiogenesis, and invasion, creating a TME that promotes tumor progression while revealing potential metabolic targets for therapeutic intervention.

Taken together, the molecular pathways that drive metabolic reprogramming in cancer cells contribute to immune evasion and the sustained growth of malignant cells. These alterations promote the establishment of an acidic microenvironment that reinforces a feedback loop within the TME, progressively shifting conditions in favor of tumor survival while impairing effective immune cell activity. Within this context, targeting these metabolic adaptations has emerged as an important therapeutic strategy, particularly as interventions aimed at restoring metabolic balance may help disrupt tumor-promoting conditions. Among the approaches currently being explored, phytochemicals have attracted attention for their capacity to influence key metabolic pathways involved in cancer progression. The following section examines the mechanisms through which phytochemicals may modulate cancer metabolism and potentially counteract the metabolic adaptations that support tumor development.

## 4. Potential Mechanisms of Phytochemicals in Modulating Altered Cancer Metabolic Pathways

Phytochemicals have been widely reported to regulate cancer cell growth and survival across multiple models, highlighting their potential as anticancer agents. Studies in both in vitro and in vivo models have quantified the effects of representative phytochemicals on tumor growth, dosing, and safety, as summarized in Table 1, with additional classification details included in Supplementary Table S1. These compounds can also intercept altered metabolic pathways, modulating redox homeostasis, nucleotide synthesis, lipid metabolism, and glycolytic flux [52,53]. The following sections examine how these compounds influence altered metabolic pathways in cancer, with a focus on glycolysis and mitochondrial function, including the PPP, the TCA cycle, and glutamine addiction.

### 4.1. Polyphenols Targeting Glycolysis

Polyphenols have shown the capacity to regulate key enzymes, transporters, and signaling pathways involved in the Warburg effect. For instance, Cervantes et al. [59] used purified yeast HK isoforms, *Saccharomyces cerevisiae* HK1 and HK2, in vitro to assess enzyme activity in the presence of resveratrol, epigallocatechin gallate, pterostilbene, and phloretin. The study found that most of the compounds reduced HK activity by more than fifty percent, with epigallocatechin gallate and certain acyl-glucoside derivatives of phloretin exhibiting the strongest inhibition. Complementary molecular docking studies indicated that similar inhibitory interactions could occur with human HK2, suggesting that these polyphenols may have the potential to modulate glycolysis in cancer cells [59]. Jia et al. [60] demonstrated that quercetin can decrease glucose uptake and lactate production while downregulating key glycolytic enzymes, including PKM2 and lactate dehydrogenase A (LDHA). Quercetin treatment also reduced the acidity of the TME providing further evidence that polyphenols have the potential to target glycolytic pathways in cancer [60]. More importantly, Chen et al. [61] investigated quercetin in erlotinib-resistant human tongue squamous carcinoma HSC-3 cells. Two resistant lines, ERL-R5 and ERL-R10, were generated by prolonged exposure to the endothelial growth factor targeting tyrosine kinase inhibitor erlotinib, resulting in enhanced growth, elevated Akt/ERK phosphorylation, and suppressed p21 and p27 expression. Quercetin at 5 μM inhibited cell viability, induced G2-phase arrest, restored p21 and p27, and reduced glucose uptake by downregulating GLUT1, PKM2, and LDHA. Mechanistically, PKM2 knockdown mimicked these effects, confirming this metabolic axis as a key target. In vivo, quercetin suppressed tumor growth and, when combined with erlotinib, enhanced apoptosis in resistant cells. These findings illustrate that targeting metabolic pathways with polyphenols not only has the potential to modulate glycolysis but also sensitize resistant cancer cells to existing therapies, offering a promising strategy to overcome resistance [61].

### 4.2. Polyphenols Targeting Mitochondrial Metabolic Dependencies

Polyphenols have been shown to target key metabolic enzymes and influence cancer cell energy dependencies. For example, Riaz et al. [62] examined pomegranate peel extract and found that it strongly inhibits 6-phosphogluconate dehydrogenase (6PGD, IC_50_ = 0.090 µg/mL), acting as a competitive inhibitor of NADP^+^ and 6-phosphogluconate binding. In silico analysis identified procyanidin, delphinidin, and cyanidin as the most active constituents, each forming stable interactions within the 6PGD active site. Consistent with this enzymatic inhibition, the extract reduced MCF-7 breast cancer cell viability (IC_50_ = 3.138 µg/mL), and acute toxicity studies in mice confirmed that it was well tolerated [62]. Similarly, Mele et al. [63] demonstrated that the polyphenol polydatin inhibits G6PD activity, resulting in NADPH depletion, ROS accumulation, and ER stress-mediated cell death [63]. Wu et al. [64] also showed that kaempferol suppresses the non-oxidative arm of the PPP through microRNA regulation in colorectal cancer cells. The treatment reduced TKT and transaldolase expression, depleted nucleotide pools, and triggered marked DNA damage, as evidenced by phosphorylated histone H2AX (γH2AX) accumulation, comet tail formation, and activation of checkpoint kinase 1 & 2 signaling. Supplementation with exogenous deoxynucleotide triphosphates rescued this phenotype, confirming that impaired ribose 5-phosphate availability underlies the replication stress. Kaempferol induces replication stress by limiting ribose 5-phosphate availability. Mechanistically, it upregulates microRNAs such as miR-195 and miR-497, which directly downregulate 6-phosphofructo-2-kinase/fructose-2,6-biphosphatase 4 (PFKFB4), a key regulator of non-oxidative PPP flux. Restoring PFKFB4 expression or inhibiting these microRNAs reverses the metabolic and DNA-damage effects, confirming that kaempferol restricts nucleotide biosynthesis through the miR-195/497-PFKFB4 axis [64].

Polyphenols have also been reported to modulate the TCA cycle, a central hub of mitochondrial energy metabolism and biosynthetic precursor. A study showed that a therapeutic combination of metformin and caffeic acid (CA) on cervical cancer cells impaired TCA-cycle anaplerosis through CA-mediated downregulation of GLS, accompanied by suppressed malic enzyme 1-dependent NADPH production stress and apoptosis [65]. Peeters et al. [66] illustrated a similar metabolic effect using epigallocatechin-3-gallate in colorectal cancer cell lines in combination with a radiotracer, showing reduced radiolabeled carbon incorporation into downstream TCA intermediates. This radiotracing evidence illustrates the capacity of polyphenols to attenuate central carbon flux and highlights their potential within theragnostic strategies, where metabolic modulation can be coupled with functional imaging to both monitor metabolic responses and enhance therapeutic efficacy [66]. Another potential benefit of using polyphenols in regulating cancer was demonstrated by Cheng et al. [67], who investigated malvidin-3-galactoside, a blueberry-derived polyphenol previously shown to suppress hepatocellular carcinoma. The study analyzed HCC mouse models’ fecal samples to assess microbiota changes and found that malvidin-3-galactoside increased beneficial butyrate-producing bacteria, reduced pathogenic species, and restored microbial metabolic activity associated with the TCA cycle. These findings suggest that polyphenols can influence the gut-tumor metabolic axis in a way that supports both tumor control and a healthier systemic metabolic environment, adding an important complementary benefit to their direct anticancer effects [67]. One challenge in using plant-based compounds is their limited bioavailability. Jiang et al. [68] addressed this by conjugating to triphenylphosphonium (TPP), enabling direct mitochondrial targeting in breast cancer cells. TPP-resveratrol conjugate enhanced the cytotoxicity of resveratrol in both human and murine breast cancer cell lines, induced greater mitochondrial membrane potential loss, and triggered higher rates of apoptosis. Metabolomic analyses revealed that TPP-resveratrol disrupted amino acid, TCA cycle, purine, and pyrimidine metabolism, effectively down-regulating energy and biosynthetic pathways essential for cancer cell proliferation. These findings demonstrate that conjugating polyphenols to mitochondrial-targeting molecules could significantly improve their anticancer efficacy by directly interfering with cancer cell metabolism [68].

**Figure 4:**
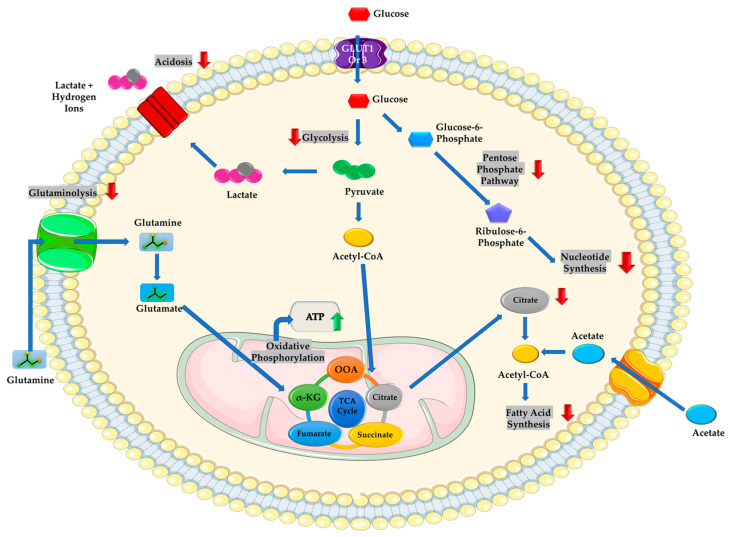
Phytochemical modulation of cancer metabolic reprogramming. Phytochemicals reprogram cellular metabolism, suppress biosynthetic pathways, and enhance mitochondrial function. Red arrows indicate suppression, while green arrows signify enhancement of metabolic processes. Glycolysis is downregulated, reducing glucose uptake via glucose transporter 1 (GLUT1), as well as pyruvate and lactate production, which lowers acidosis. The pentose phosphate pathway (PPP) is inhibited, decreasing ribulose-5-phosphate formation and nucleotide synthesis, thereby limiting rapid proliferation. Glutaminolysis, the conversion of glutamine to glutamate and its entry into the tricarboxylic acid (TCA) cycle and fatty acid synthesis are also suppressed, reducing glutamine-driven anaplerosis and lipid biosynthesis. Conversely, oxidative phosphorylation and the TCA cycle are enhanced, promoting adenosine triphosphate (ATP) production, restoring redox balance, and ensuring efficient energy generation. Key intermediates shown include alpha-ketoglutarate (α-KG), oxaloacetate (OAA), fumarate, succinate, and citrate. Acetyl coenzyme A (Acetyl-CoA) links glycolysis and lipid metabolism. These metabolic shifts create an unfavorable environment for cancer cell survival, thereby supporting cellular health and longevity. This figure summarizes the overall metabolic landscape influenced by phytochemicals as presented throughout the review.

Beyond their direct effects on tumor metabolic pathways, polyphenols may also modulate amino acid metabolism and nutrient-sensing pathways, potentially influencing cellular amino acid availability and utilization, including glutamine-dependent metabolic processes. Glutamine serves as a central carbon and nitrogen donor in cancer cells, alterations in amino acid metabolism can further amplify glutamine addiction and sustain key downstream pathways such as glycolysis, glutaminolysis, and one-carbon metabolism (Figure 4) [39]. For instance, xanthohumol was shown to inhibit cellular glutamine uptake in breast cancer cell lines through this metabolic axis, demonstrating its ability to modulate glutamine-dependent metabolic fluxes [69]. Accordingly, Yu et al. [70] demonstrated that *Aronia melanocarpa* elliot anthocyanins suppress the development of colon cancer by reducing the expression of GLS and the glutamine transporter SLC1A5, lowering mTORC1 phosphorylation, and attenuating pro-inflammatory cytokine signaling [70]. In drug-resistant cancer, curcumin has been shown to counteract glutamine-linked metabolic adaptations that drive multidrug resistance. Zhang et al. [71] demonstrated that curcumin reverses P-glycoprotein mediated doxorubicin resistance in SW620/Ad300 colon cancer cells by suppressing D-glutamine metabolism, a pathway markedly upregulated in resistant cells. Curcumin reduced the intracellular ATP required for P-glycoprotein efflux activity, thereby compromising its function. Metabolomic profiling further showed that curcumin diminished polyamine biosynthesis through downregulation of ornithine decarboxylase, weakening the glutamine-derived metabolic network that sustains antioxidant capacity and drug efflux. Disrupting these glutamine-dependent resistance circuits, curcumin restored intracellular doxorubicin accumulation and re-sensitized resistant cells to apoptosis [71]. The therapeutic exploitation of glutamine addiction in drug-resistant cancers was further demonstrated by Dai et al. [72], who developed a metal-polyphenolic nanomedicine incorporating the polyphenol epigallocatechin gallate, the GLS1 inhibitor bis-2-(5-phenylacetamido-1,2,4-thiadiazol-2-yl) ethyl sulfide (BPTES), and doxorubicin. Their multifunctional nanomedicine simultaneously inhibited glutamine metabolism, depleted glutathione, and amplified ROS-induced stress, thereby weakening the metabolic resilience conferred by glutamine addiction. As a result, the nanomedicine markedly sensitized pancreatic cancer cells and patient-derived organoids to doxorubicin-induced DNA damage and chemodynamic therapy, effectively overcoming glutamine-driven chemoresistance [72].

### 4.3. Terpenes and Terpenoids Targeting Glycolysis

**Figure 5:**
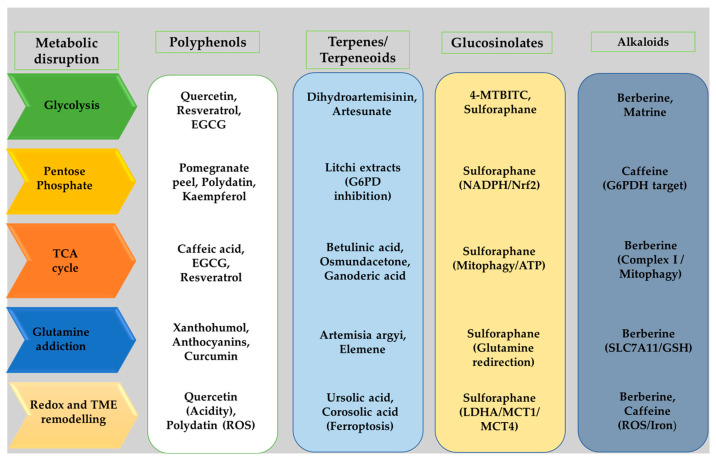
Phytochemical classes disrupt cancer metabolic dependencies. Plant-derived phytochemical classes (polyphenols, terpenes/terpenoids, glucosinolates, and alkaloids) target interconnected metabolic pathways that sustain tumor growth. These compounds interfere with glycolysis, the pentose phosphate pathway, tricarboxylic acid (TCA) cycle, redox balance, tumor microenvironment remodeling, and glutamine addiction. Mechanistically, phytochemicals inhibit key metabolic enzymes, including lactate dehydrogenase A (LDHA), glucose-6-phosphate dehydrogenase (G6PD), and glutaminase (GLS). They also modulate nicotinamide adenine dinucleotide phosphate (NADPH) production and redox homeostasis, disrupt mitochondrial oxidative phosphorylation, and alter TCA-cycle anaplerosis. Additional targets include nuclear factor erythroid 2-related factor 2 (Nrf2), lactate dehydrogenase A (LDHA), and monocarboxylate transporters 1and 4 (MCT1/MCT4), as well as solute carrier family 7 member 11 (SLC7A11) involved in glutathione metabolism. The distribution of compounds across these pathways highlights the multifaceted nature of phytochemical-mediated metabolic disruption and its potential to weaken chemoresistance-associated adaptations and enhance therapeutic sensitivity.

Terpenes and terpenoids also have the potential to modulate cancer metabolism by disrupting glycolytic flux and associated proliferative signaling. In a presurgical breast cancer trial, 2 g/day limonene for 2–6 weeks altered key glycolysis-related metabolites, raising pyruvate, fructose, and glucuronate while lowering acetylcarnitine. These coordinated metabolic and signaling shifts show that terpenoids can interfere with cancer energy pathways to suppress tumor growth [73]. They either target enzymes directly involved in the Warburg effect or modify the availability of critical components that sustain this metabolic shift (Figure 5). For example, Zhang et al. [74] reported that the artemisinin derivatives dihydroartemisinin and artesunate reduce the expression of GLUT1, HK, and LDH, accompanied by decreased glucose uptake and ATP production [74]. In chemoresistant cancer cells, terpenes and terpenoids have the capacity to reverse resistance-associated metabolic changes and help restore the effectiveness of anticancer drugs. Lou et al. [75] demonstrated this using ursolic acid in doxorubicin-resistant breast cancer cells. Ursolic acid regulated ATP production, suppressed aerobic glycolysis, and effectively inhibited mitochondrial respiration [75].

### 4.4. Terpenes and Terpenoids Targeting Mitochondrial Metabolic Dependencies

Terpenes and terpenoids have shown potential in regulating mitochondrial metabolic dependencies as illustrated by Kanwal et al. [76] who investigated methanolic extracts from various parts of *Litchi chinensis* (leaves, bark, and seeds) for their effects on G6PD activity in liver cancer (HepG2) cells. The extracts demonstrated potent inhibition of G6PD, with IC_50_ values of 1.199 μg/mL (leaf), 2.350 μg/mL (bark), and 1.238 μg/mL (seeds). This enzymatic inhibition translated into a significant reduction in HepG2 cell viability, while acute toxicity studies in mice confirmed the extracts were well tolerated. Mechanistically, targeting G6PD limits NADPH availability, thereby impairing redox homeostasis and increasing oxidative stress selectively in cancer cells. These findings indicate the potential of terpenoid-rich plant extracts to disrupt PPP-dependent metabolic support [76]. Similarly, Yang et al. [77] investigated ginseng-derived nanoparticles (GDNPs) and their effects on non-small cell lung cancer (NSCLC) cells. Functionally, GDNPs suppressed migration, invasion, adhesion, and colony formation of NSCLC cells in a dose-dependent manner. Mechanistically, GDNPs also inhibited epithelial-to-mesenchymal transition, increasing E-cadherin and decreasing vimentin expression. These findings highlight that terpenoid-rich plant-derived nanoparticles can influence multiple cancer-associated metabolic and signaling pathways, including those connected to glucose utilization and cellular redox states, supporting their potential as adjunctive anticancer agents [77].

Terpenes and terpenoids have also been shown to target the TCA cycle. Guerra et al. [78] investigated the effects of betulinic acid (BA) and ursolic acid (UA) in triple-negative breast cancer cell lines, demonstrating that BA markedly altered glucose metabolism by enhancing TCA-cycle activity and promoting the hydrolysis of neutral lipids, whereas UA exerted only moderate metabolic effects. In non-tumorigenic MCF-10A breast epithelial cells, treatment with these terpenoids increased glucose flux through the TCA cycle and diverted glycolytic intermediates toward the hexosamine biosynthetic pathway and neutral lipid synthesis, possibly stored in lipid droplets as a detoxification mechanism. Additionally, pyruvate utilization and TCA activity were intensified, potentially compensating for reduced glycolytic pyruvate production due to oxidative stress [78]. Similarly, Jin et al. [79] demonstrated that osmundacetone, a naturally occurring terpenoid, inhibits mitochondrial serine hydroxymethyltransferase (SHMT2), a key enzyme that supports anaplerotic flux into the TCA cycle under metabolic stress. Osmundacetone treatment suppressed SHMT2 expression, reduced mitochondrial membrane potential, and decreased oxygen consumption rates, indicating impaired oxidative phosphorylation. Notably, the compound also downregulated fatty acid β-oxidation, limiting acetyl-CoA availability and further restricting TCA-cycle activity. This dual inhibition, blocking both SHMT2-mediated anaplerosis and fatty acid oxidation-derived acetyl-CoA supply, effectively reduced mitochondrial metabolism and weakened the cancer cells’ energy supply [79].

The metabolic impact of terpenoids on mitochondrial pathways is further supported by recent preclinical evidence by Lei et al. [80] who demonstrated that ganoderic acid T (GAT) derived from *Ganoderma lucidum*, effectively suppresses hepatocellular carcinoma proliferation and metastasis across multiple experimental models, including HepG2, Hep3B, Huh7, LM3, SNU449, and MHCC-97H cell lines, as well as nude mouse xenografts. Using complementary biochemical and molecular assays, GAT was shown to directly bind pyruvate carboxylase, an anaplerotic enzyme responsible for converting pyruvate into oxaloacetate to sustain TCA-cycle flux. This interaction occurs through an allosteric mechanism that enhances pyruvate carboxylase enzymatic activity, leading to metabolic disruption rather than reinforcement of TCA cycling. Enhanced pyruvate carboxylase activity led to excessive oxaloacetate accumulation, mitochondrial oxidative phosphorylation dysfunction, elevated ROS, and activation of ROS-JNK/p38 MAPK stress pathways. This maladaptive metabolic reprogramming impaired mitochondrial efficiency, reduced energy production, and inhibited metastatic phenotypes [80]. Another important mechanism involves phytochemical-mediated sensitization of chemotherapeutic agents through modulation of drug-efflux transporters and survival pathways. Caryophyllane sesquiterpenes have demonstrated this effect in hepatocellular carcinoma, cholangiocarcinoma, and pancreatic ductal adenocarcinoma models. Combined use of these compounds with sorafenib resulted in significant downregulation of multidrug resistance transporters. They further reported a suppression of STAT3 activation, a pathway closely associated with drug resistance, metabolic adaptation, and cell motility. In their models, inhibition of the STAT3/ABC-transporter axis corresponded with reduced migratory capacity and a marked increase in sorafenib efficacy. These findings reinforce the emerging concept that certain terpenoids can reprogram metabolic-signaling networks to reverse chemoresistance, affecting not only mitochondrial or TCA-linked pathways but also drug-efflux systems that contribute to poor therapeutic response [81].

Terpenes and terpenoids have also shown potential in targeting glutamine addiction in cancer cells. A study by Zhang et al. [82] demonstrated the potential to regulate glutamine metabolism using *Artemisia argyi* in a pancreatic cancer model, showing that this compound suppressed tumor growth by reprogramming glutamine metabolism and promoting ferroptosis [82]. Similarly, Song et al. [83] used elemene to inhibit glutathione synthesis in lung adenocarcinoma, showing that it reduces GSH levels and the GSH/GSSG ratio by downregulating GLS, SLC7A11, and glutathione synthase (GST), ultimately disrupting redox homeostasis and promoting apoptosis [83]. Wu et al. [84] demonstrated that ursolic acid restored cisplatin sensitivity in HepG2/DDP hepatocellular carcinoma cells by reducing the elevated IC_50_, enhancing apoptosis, and correcting mitochondrial dysfunction. This re-sensitization was associated with suppression of the Nrf2/ARE pathway, including downregulation of heme oxygenase-1, NAD(P)H quinone dehydrogenase 1, and GSTs, thereby weakening the antioxidant defenses that maintain the chemoresistant phenotype [84]. A similar study by Lin et al. [85] reported that corosolic acid enhanced cisplatin sensitivity in gastric cancer, showing that a low dose of corosolic acid (5 µM) was non-toxic to normal gastric epithelial cells but significantly reduced viability in AGS and MKN-45 cells, and its combination with cisplatin further decreased viability in both parental and cisplatin-resistant AGS-CR cells. The combined treatment also reduced colony-forming capacity, increased apoptosis, and more effectively suppressed tumor growth in xenograft models without affecting body weight. Mechanistically, corosolic acid promoted ferroptosis, increasing intracellular iron, ROS, and lipid peroxidation while lowering GSH levels. This effect was linked to marked suppression of glutathione peroxidase 4, and the chemosensitizing action of corosolic acid was reversed by ferroptosis inhibition or glutathione peroxidase 4 overexpression. Together, these findings indicate that corosolic acid overcomes cisplatin resistance by driving glutathione peroxidase 4-dependent ferroptosis [85].

### 4.5. Glucosinolates Targeting Glycolysis

Glucosinolates and their bioactive derivatives have shown the potential of modulating key glycolytic pathways in cancer cells, disrupting the energy production essential for tumor growth. Singh et al. [86] demonstrated that 4-(methylthio) butyl isothiocyanate (4-MTBITC), derived from glucoerucin, exerted anticancer effects in 7,12-dimethylbenz [a]anthracene (DMBA) induced mammary carcinoma in rats. Treatment with 4-MTBITC normalized the DMBA-induced alterations in body weight and feed intake, improved hepatic, renal, and lipid profiles, and enhanced antioxidant defenses by increasing superoxide dismutase and glutathione while reducing malondialdehyde and hydroxyl radicals. At the molecular level, 4-MTBITC downregulated the overexpression of glycolytic enzymes, including HK, phosphoglucose isomerase, glyceraldehyde-3-phosphate dehydrogenase, enolase, and PK, and attenuated lactate production. It also modulated hypoxia-related signaling by reducing HIF-1α and mTOR expression. Furthermore, 4-MTBITC restored the balance of key amino acids such as serine, arginine, alanine, asparagine, glutamate, and tryptophan, and influenced protein markers involved in and apoptosis, including Akt, mTOR, p21, p53, and nuclear factor kappa-light-chain-enhancer of activated B (NF-kB). Histopathological analysis confirmed reduced neoplastic infiltration following treatment. These results suggest that 4-MTBITC modulates glycolytic flux, hypoxia signaling, and amino acid metabolism to counteract tumor progression, highlighting its potential role in targeting metabolic reprogramming in cancer cells [86]. Similarly, Huang et al. [87] investigated SFN, a bioactive derivative of glucoraphanin, to examine its effects on glycolytic pathways. SFN treatment inhibited ATP production and oxidative phosphorylation, notably suppressing the activity of key metabolic enzymes including HK2 and PDH [87]. Another study highlighted the effects of SFN on lactate metabolism, specifically, Shi et al. [88] identified SFN as a novel LDHA inhibitor that suppresses NSCLC metastasis in the acidic TME. SFN upregulates microRNA-7-5p (miR-7-5p), which inhibits c-Myc, resulting in decreased LDHA expression and reduced lactate production. Additionally, SFN modulates MCT1 and MCT4, thereby impairing lactate transport and the metabolic adaptation of cancer cells [88].

### 4.6. Glucosinolates Targeting Mitochondrial Metabolic Dependencies

Several studies have investigated the impact of glucosinolates on mitochondrial metabolic function (Figure 5). For instance, Bernuzzi et al. [89] demonstrated that SFN disrupts mitochondrial metabolism and reduces ATP production in HepG2 cells. Consistent with these findings, exposure to SFN in different metabolic conditions revealed widespread remodeling of energy metabolism. In high-glucose (25 mM) conditions, SFN decreased TCA cycle intermediates such as citrate and succinate, while basal (5.5 mM) glucose conditions showed more modest effects. SFN also upregulated genes involved in mitochondrial redox balance, suggesting enhanced antioxidant capacity. Metabolic tracing indicated that SFN limits glutamine entry into the TCA cycle, redirecting it toward glutathione biosynthesis, while simultaneously reducing serine and glycine pools by downregulating phosphoglycerate dehydrogenase and glycine decarboxylase. Additionally, SFN promoted NADPH production through increased PPP activity, supporting antioxidant reactions and maintaining redox homeostasis. Importantly, Nrf2 knockdown abolished these effects, confirming that SFN modulates mitochondrial and central carbon metabolism primarily through Nrf2-dependent mechanisms. Together, these findings suggest that glucosinolate derivatives may not only induce antioxidant responses but also influence mitochondrial energy metabolism, glutamine utilization, and redox balance, particularly under conditions of metabolic stress [89]. Plafker et al. [90] used the same phytochemical to demonstrate how SFN mimics nutrient deprivation by preserving mitochondrial function, promoting mitophagy and lysosomal biogenesis, suppressing mTOR signaling, enhancing autophagic flux, and reprogramming glucose metabolism. Specifically, they found that SFN increased mitochondrial mass and maintained membrane potential, improved the redox balance of the mitochondrial matrix, and induced mitochondrial hyperfusion, indicating enhanced organelle integrity. Additionally, SFN promoted the selective turnover of damaged mitochondria via mitophagy, reduced glycolytic flux, and shifted pyruvate metabolism toward oxidative phosphorylation, thereby activating key cellular starvation responses without actual nutrient restriction [90].

### 4.7. Alkaloids Targeting Glycolysis

In regulating the Warburg effect, berberine, for example, modulates HK2, PFK, and LDHA, while also disrupting metabolic reprogramming through HIF-1α modulation (Figure 5) [91]. Similarly, matrine reverses the Warburg effect by reducing glucose uptake and lactate production, downregulating HIF-1α. It also targets GLUT1, HK2, and LDHA, ultimately suppressing tumor growth [92]. Wang et al. [93] showed that matrine alkaloids can resensitize cisplatin-resistant NSCLC cells (A549/DDP) by inhibiting DNA damage repair and disrupting survival pathways that sustain metabolic adaptation. Functionally, matrine treatment reduced proliferation, migration, and invasion, induced G0/G1 arrest, and significantly increased apoptosis, accompanied by elevated BAX and cleaved caspase-3 and reduced Bcl-2 expression. Crucially, matrine downregulated DNA repair protein RAD51 homolog 1 (RAD51), DNA polymerase delta subunit 2 (POLD2), and phosphorylated histone H2AX (H2A histone family member X) (γ-H2AX), indicating impaired repair of cisplatin-induced damage. In vivo, matrine markedly reduced tumor growth in A549/DDP xenografts and decreased RAD51, POLD2, and γ-H2AX expression while enhancing apoptotic signaling. Together, these results show that matrine alkaloids overcome cisplatin resistance by disrupting DNA repair capacity and disabling the metabolic advantage that resistant cells acquire, positioning matrine as a promising glycolysis-modulating chemosensitizer [93].

### 4.8. Alkaloids Targeting Mitochondrial Metabolic Dependencies

Alkaloids can be used to reprogram mitochondrial metabolism, for instance, Mori et al. [94] showed that berberine potently rewires mitochondrial bioenergetics in gastrointestinal cancer models. Berberine produced concentration-dependent cytotoxicity (48 h IC_50_: CT26, murine colon carcinoma, 17.2 μM; HT29, human colorectal adenocarcinoma, 11.9 μM; TMK-1, human gastric carcinoma, 9.7 Μm), reduced invasion and sphere formation, and sensitized cells to 5-fluorouracil (5-FU IC_50_ reduced by 10–33%). At the organelle level, BBR increased mitochondrial oxidative stress (mitochondrial superoxide and mitochondrial Fe^2+^), elevated lipid peroxidation, and reduced mitochondrial membrane potential. Metabolically, BBR suppressed basal oxygen consumption rate and modestly lowered ATP while paradoxically increasing glycolytic flux, accompanied by downregulation of c-Myc and upregulation of several metabolic enzymes (PKM, malic enzyme 1, acetyl-CoA carboxylase alpha, G6PD). Mechanistically, berberine inhibited mitochondrial complex I activity (with a compensatory rise in complex II activity), induced mitophagy and autophagy-related gene 5-dependent -dependent autophagy, and promoted ferroptosis-linked changes (decreased GSH, SLC7A11, and glutathione peroxidase 4) [94].

Treatment with pharmacological inhibitors, including ferrostatin-1, deferoxamine, caspase inhibitors, and malonate, revealed that complex I inhibition, mitophagy, and ferroptosis all contribute to BBR-mediated cell death. Finally, in vivo berberine markedly suppressed the peritoneal dissemination of CT26 cells while exhibiting limited toxicity. The results show that berberine remodels mitochondrial function, increases mitochondrial ROS/iron and mitophagy, inhibits complex I, and engages ferroptotic and apoptotic programs, a multifaceted mitochondrial reprogramming that impairs tumor fitness and chemosensitivity [94]. Alkaloids also directly impact enzymes in the PPP, TCA cycle, and glutamine metabolism. Xu et al. [95] demonstrated this effect by showing that the xanthine alkaloid caffeine inhibits renal cell carcinoma proliferation through targeting G6PDH, the rate-limiting enzyme of the PPP. In vitro, caffeine directly bound to G6PDH with high affinity, reduced its coenzyme (NADP^+^) and substrate (G6P) binding, and inhibited dimer formation, which resulted in concentration-dependent suppression of cell viability and colony formation, as well as induction of apoptosis. Mechanistically, caffeine disrupted G6PDH-mediated redox homeostasis, reducing NADPH levels and ROS accumulation while modulating antioxidant proteins. Caffeine also inhibited the p-STAT3/cyclin D1 axis, further impairing cell proliferation. In vivo, caffeine treatment significantly decreased tumor growth in xenografts models, suppressed G6PDH activity in tumor tissues, reduced Kiel 67 antigen and p-STAT3 levels, and altered redox-related protein expression, without affecting body weight. These findings highlight that caffeine directly targets G6PDH to disrupt PPP flux, redox balance, and proliferative signaling, demonstrating a potent mechanism for renal cell carcinoma suppression [95]. Overall, these studies show that phytochemicals exert multifaceted anticancer effects by targeting central nodes of tumor metabolism, including glycolysis, mitochondrial function, the PPP, glutamine dependence, and broader TME-related metabolic interactions. By modulating metabolic enzymes, redox homeostasis, hypoxia-driven pathways, and angiogenic signaling, alkaloids such as berberine, matrine, caffeine, cryptolepine, and solanidine disrupt the metabolic programs that support cancer cell survival, proliferation, and adaptive stress responses.

### 4.9. Phytochemicals Targeting the Tumor Microenvironment

The TME is a highly heterogeneous and dynamic system shaped not only by diverse cellular components, including mesenchymal cells, immune cells, circulating tumor cells, cancer stem cells, and endothelial cells, but also by the underlying metabolic reprogramming of cancer cells [96]. These metabolic alterations, such as enhanced glycolysis, glutamine dependence, and redox imbalance, play a central role in defining the biochemical landscape of the TME, contributing to features such as nutrient competition, hypoxia, and extracellular acidification. Phytochemicals from various classes have demonstrated the capacity to modulate these metabolically driven conditions within the TME. Beyond inducing direct cytotoxic effects, these compounds can interfere with key metabolic pathways that regulate lactate production, pH balance, and redox homeostasis, thereby disrupting the tumor-supportive environment [97]. Since cancer metabolism is a major determinant of TME acidification and immune suppression, targeting these pathways with phytochemicals presents a promising strategy to indirectly reshape the TME and impair tumor progression. For instance, Jiménez et al. [98] evaluated a standardized polyphenol-rich extract (P2Et) derived from *Caesalpinia spinosa* and demonstrated its capacity to disrupt key metabolic and functional features of cancer-associated fibroblasts (CAFs) within the TME. Specifically, P2Et counteracted TGFβ1-induced CAF differentiation by restoring caveolin-1 expression, reducing glucose uptake, and attenuating ROS production. Functionally, this metabolic interference translated into a diminished ability of CAFs to support tumor cell clonogenicity, migration, and epithelial-mesenchymal transition (EMT), as well as a reduction in cancer stem cell-transcriptional programs [98]. In regulating hypoxia and pH, Su et al. [99] demonstrated the effects of sanguinarine, an alkaloid, on the TME, noting the effective suppression of hypoxia-driven signaling pathways central to tumor adaptation. Specifically, sanguinarine suppressed hypoxia-induced expression of EphB4 and significantly reduced HIF-1α protein levels by promoting its proteasomal degradation and inhibiting its nuclear translocation, without altering its transcription. This attenuation of HIF-1α activity was further accompanied by inhibition of MAPK/ERK signaling and disruption of HIF-1α/STAT3 interactions, thereby limiting downstream transcriptional programs associated with hypoxic adaptation. Importantly, sanguinarine reduced the expression of key HIF-1α target genes, including CA9 and VEGF, which are directly implicated in extracellular acidification and angiogenesis. Functionally, these molecular effects translated into decreased tumor cell proliferation and reduced angiogenic potential both in vitro and in vivo [99]. Phytochemicals have also been shown to modulate the immune landscape within the TME, with Wang et al. [100] demonstrating that triterpenoid-rich extracts from *Rhus chinensis* regulate glucose metabolism in colorectal cancer while simultaneously restoring CD8^+^ T-cell function. Mechanistically, these extracts reduced tumor glycolysis and lactate production, alleviating metabolic competition within the TME, while enhancing T-cell metabolic fitness through activation of the Akt/mTOR pathway. This resulted in improved effector function, characterized by increased cytokine production and reduced expression of exhaustion markers such as PD-1, ultimately contributing to suppressed tumor growth in vivo [100]. The TME may be reshaped through phytochemical-mediated modulation of metabolic reprogramming, a central driver of tumor progression and immune suppression. By targeting these pathways, phytochemicals may disrupt tumor-supportive conditions while restoring anti-tumor immune function, thereby potentiating therapeutic responses.

## 5. Challenges and Limitations

Although the preclinical literature indicates that phytochemicals can modulate cancer metabolism and the TME, several important challenges hinder translation into validated clinical therapies. Phytochemicals are typically present in low concentrations in plant material, so obtaining pharmacologically relevant doses requires large quantities of biomass and extensive processing. Scale-up from bench extraction to GMP-grade material involves agricultural, botanical, and chemical inputs (cultivation, harvest timing, extraction solvents, and purification), each of which influences yield and reproducibility [53]. The purification steps required to isolate a single active compound often lead to substantial losses of material and increase production costs, limiting feasibility in resource-constrained settings. Development of phytochemical therapeutics is resource intensive: it requires coordinated teams across agronomy, analytical chemistry, formulation science, pharmacology, and regulatory affairs [101]. The time, specialized equipment, and skilled personnel required for reliable extraction, purification, and quality control create financial and logistical barriers, particularly for small research groups and low- and middle-income regions that might otherwise benefit most from affordable phytochemical approaches [102].

A key limitation of many phytochemicals is their inherently low systemic bioavailability following oral or parenteral administration. Factors such as rapid metabolic degradation, limited stability in physiological fluids, and extensive first-pass metabolism substantially diminish their circulating levels and restrict effective tissue distribution. Moreover, limited robust pharmacokinetic profiling makes it difficult to determine whether in vivo anti-tumor effects can be achieved at tolerable systemic doses [103]. The heterogeneity of tumor metabolism and inter-tumor variability further complicates predictions of clinical efficacy and selectivity. Many phytochemicals are chemically labile (oxidation, hydrolysis) or have poor aqueous solubility, which undermines stability during storage and after administration [104]. Ethical considerations, including sustainable sourcing of plant material and effects on local communities or ecosystems, also require attention during scale-up. Taken together, these practical, pharmacological, and ethical challenges highlight why translating promising phytochemical activity into safe, effective, and scalable clinical therapies remains a complex and resource-intensive endeavor.

## 6. Future Perspectives

Advances in understanding cancer metabolism continue to highlight new therapeutic opportunities. Increasingly, metabolism is being explored as a point of intervention for the development of targeted therapies that can manipulate or exploit pathways supporting tumor growth and survival [105]. Phytochemicals are gaining attention as bioactive compounds with the ability to modulate multiple cellular processes [106]. Evidence supporting this potential is reflected in the clinical success of plant-derived agents such as paclitaxel (Taxol), illustrating the translational relevance of these compounds [107]. Importantly, the concept of combination strategies in cancer therapy has been explored for decades, as reflected in earlier clinical investigations such as the Phase III Intergroup trial (E1193), which evaluated paclitaxel in combination with doxorubicin in metastatic breast cancer [108]. This highlights the longstanding clinical interest in combining agents to optimize therapeutic outcomes. Yet resistance continues to limit long-term therapeutic success, driving a shift beyond single-agent strategies toward approaches that improve efficacy while reducing toxicity. This is increasingly being investigated through combination strategies, where phytochemicals are used alongside conventional therapies to enhance treatment response. For instance, Chan et al. [61] demonstrated that pairing phytochemicals with tyrosine kinase inhibitors has potential in overcoming acquired resistance, restoring therapeutic sensitivity in cancers that had become resistant [61]. Similarly, resistance to agents such as Taxol remains a major limitation in cancer therapy, prompting investigation into adjunct strategies. In this context, curcumin has been evaluated in paclitaxel-resistant models, where it was shown to enhance drug sensitivity and reverse resistance-associated phenotypes. These effects have been linked to its ability to modulate oxidative stress and disrupt survival pathways associated with chemoresistance [109].

Beyond their mechanistic effects, phytochemicals may also contribute to improved treatment tolerability and patient quality of life. Phytochemicals demonstrate dual-action potential by exerting selective cytotoxicity against malignant cells while simultaneously conferring protective effects on normal tissues, with observational evidence linking plant-rich diets to reduced overall cancer risk and supporting their role in chemoprevention [110]. Their generally lower toxicity profiles, together with reported roles in alleviating treatment-associated side effects such as inflammation, pain, anorexia, and muscle-related symptoms, further suggest a broader therapeutic value. In clinical and supportive care contexts, agents such as *Aloe vera* and *Withania somnifera* have been used to improve cancer-related fatigue and symptom burden, while compounds like ginger and green tea polyphenols provide additional supportive benefits through anti-nausea effects and inhibition of metastatic progression [111,112,113]. For instance, Lee et al. [114] demonstrated that several phytochemicals, including magnolol, fisetin, and sclareol, attenuated cisplatin-induced muscle atrophy in vitro by reducing inflammatory signaling (IL-6) and suppressing atrophy-associated markers such as myostatin [114]. Given that cisplatin is a widely used chemotherapeutic agent capable of crossing the blood-brain barrier and contributing to systemic toxicity, these findings support the potential role of phytochemicals in mitigating treatment-associated complications, particularly in the context of cancer-related cachexia [114,115]. This reduction in toxicity may have implications beyond clinical outcomes, including the preservation of functional capacity and overall patient well-being during treatment. For example, Cho et al. [116] conducted an 8-week dietary intervention designed to increase intake of phytochemical-rich fruits and vegetables in breast cancer patients, resulting in significant increases in antioxidant nutrient intake and circulating levels [116]. While global quality-of-life scores did not change significantly over the short intervention period, the observed metabolic and nutritional improvements suggest a contributory role for phytochemical-rich dietary strategies in maintaining physiological resilience and supporting health status during and after treatment. Clinical evidence further supports the role of phytochemical-based interventions in modulating treatment-related toxicity. In a randomized, triple-blind pilot trial, Shah et al. [117] investigated a 0.1% curcumin-based mouthwash in head and neck cancer patients undergoing radiotherapy and demonstrated a significant reduction in early mucosal injury dynamics. Time-to-event analysis showed that curcumin delayed the onset of radiation-induced oral mucositis by approximately two weeks, with a 50% reduction in the hazard of onset compared with benzydamine after adjustment for clinical confounders, indicating a substantially lower instantaneous risk during the early treatment phase. Despite, methodological limitations including sample attrition and a small cohort size, these findings suggest that curcumin may meaningfully reduce acute treatment-related symptom burden by delaying the onset of mucosal toxicity, thereby potentially improving tolerance to radiotherapy [117]. This illustrates the multifunctionality of phytochemical interventions that may extends beyond direct anticancer effects to include modulation of metabolic pathways, reduction in treatment-related toxicity, and support of overall physiological resilience during cancer therapy.

However, translating these benefits into consistent therapeutic outcomes remains dependent on improving the delivery and bioavailability of phytochemicals. Efforts to overcome limitations such as poor bioavailability have led to the development of advanced delivery and optimization strategies, including nanoparticle systems, vesicle-mediated transport, and drug conjugation. As illustrated by Dai et al., nanodelivery platforms enhance solubility, tumor penetration, and targeted delivery to metabolically active niches within the TME [72]. Another example of this is provided by Peng et al. [118], who investigated a nanoparticle-based delivery system for resveratrol loaded into layered double hydroxides (LDHs), demonstrating enhanced anticancer efficacy through the inhibition of glycolysis in breast cancer models [118]. These systems can be engineered to exploit pH gradients, receptor overexpression, or metabolic signatures characteristic of metastatic disease. Ongoing work further suggests that nanostructures incorporating phytochemicals may effectively modulate tumor metabolism in situ, opening avenues for interventions that target highly aggressive or metastatic cancers through microenvironment-responsive delivery [16]. Integration with radiotracing and computational approaches is further improving the precision and feasibility of these interventions [119]. In parallel, the expanding use of radiolabeled tracers offers new opportunities for real-time monitoring of phytochemical distribution and metabolic impact [120]. Metabolic shifts in cancer cells generate distinct molecular profiles that differentiate malignant tissues from their normal counterparts. Advances in cancer imaging have leveraged these metabolic alterations to develop novel radiotracers, molecular imaging modalities, and targeted radionuclide therapies. These tools exploit the dysregulated metabolic landscape of tumors to enhance diagnostic accuracy, monitor disease progression, and inform precision treatment strategies [121]. By targeting tumor-specific metabolic processes such as elevated glucose uptake, increased choline turnover, and amino acid metabolism, functional imaging provides critical insights that go beyond structural evaluation, allowing for earlier detection, improved staging, and real-time monitoring of therapeutic response [122]. Table 2 summarizes radiolabeled agents used in imaging tumor metabolism, outlining their specific metabolic targets and clinical utility in cancer detection.

These developments extend beyond diagnostic utility, providing a foundation for integrating phytochemicals into combination treatment strategies alongside established therapeutic modalities. Coupling phytochemicals to radiotracers, or co-administering them with metabolic tracers, as demonstrated by Peeters et al. [66], could enable simultaneous mapping of drug biodistribution and tumor metabolic response [66]. Such strategies would support precision dosing, early detection of therapeutic effect, and improved identification of responders versus non-responders. Although safety considerations remain, these radiotracer-phytochemical integrations align closely with the broader movement toward precision oncology. Beyond pharmacological combinations, phytochemicals may also potentiate established treatment modalities such as radiotherapy and immunotherapy through modulation of metabolic reprogramming and the TME. However, these applications remain largely underexplored, particularly in clinical settings.

A critical next step in advancing this field is the translation of phytochemicals from preclinical models into patient-based studies to validate their overall therapeutic impact. While preclinical evidence consistently demonstrates their ability to modulate key metabolic pathways, this has not yet been adequately reflected in clinical trial design. As shown in Table 3, existing studies incorporating phytochemicals are often not specifically designed to assess metabolic endpoints, but rather focus on conventional clinical outcomes. This highlights a disconnect between mechanistic insights and clinical evaluation. Future studies should therefore prioritize the integration of metabolic biomarkers, pathway-specific readouts, and patient stratification strategies to better capture the functional impact of phytochemicals in vivo. Addressing this gap will be essential for establishing their clinical relevance and positioning them within modern, metabolism-targeted therapeutic frameworks.

The rise of AI-assisted metabolomics and computational biology adds another layer of future potential [131]. Machine learning tools are increasingly used to characterize metabolic pathways, identify binding pockets, and predict phytochemical-target interactions across diverse cancer types. These models can prioritize candidate compounds for specific metabolic phenotypes, streamline drug design, and reduce the empirical burden of trial-and-error screening [132]. When combined with high-dimensional metabolomics and structural prediction algorithms, AI systems can also support preclinical model design, enabling in silico simulations of treatment response, resistance evolution, and synergistic combinations with existing chemotherapies or targeted agents [132].

**Figure 6:**
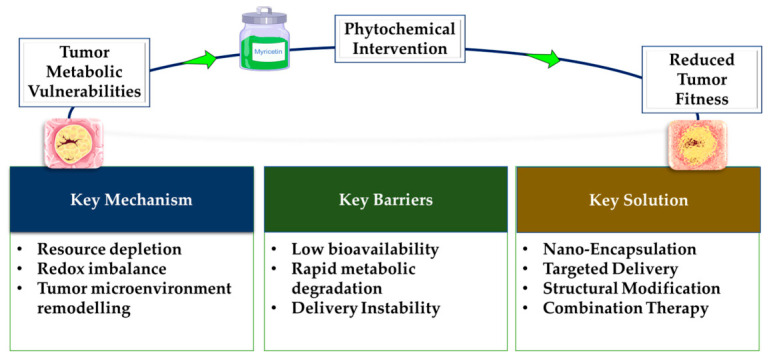
Phytochemical Modulation of Tumor Metabolic Fitness and Strategies for Clinical Translation. Conceptual summary of how phytochemicals regulate tumor growth by targeting metabolic vulnerabilities. These compounds disrupt key processes that sustain tumor fitness, including resource acquisition, redox balance, and tumor microenvironment remodeling, ultimately reducing metabolic adaptability and proliferative capacity. Despite promising mechanistic effects, clinical translation is limited by low bioavailability, rapid metabolic degradation, and delivery instability. Emerging strategies such as nano-encapsulation, targeted delivery systems, structural optimization, and rational combination therapies are proposed to enhance therapeutic exposure and improve translational potential. This framework integrates mechanistic insight with practical considerations for advancing phytochemical-based metabolic interventions in cancer.

Phytochemicals hold significant promise as next-generation therapeutic agents for cancer patients. Beyond their ability to modulate key metabolic pathways, their generally lower toxicity and reduced side effects are particularly noteworthy. However, continued research is needed to explore their standalone efficacy, as well as their potential in combination with established chemotherapeutics, including chemoresistant agents. Integrating phytochemicals with advanced strategies such as radiotracing, radiolabeling, and AI-guided approaches may further optimize treatment precision and effectiveness, enabling more personalized and targeted cancer therapies. Collectively, these strategies highlight the potential of phytochemicals to complement existing interventions while addressing treatment resistance and improving patient outcomes. As illustrated in Figure 6, their therapeutic potential lies not only in disrupting tumor metabolic fitness but also in overcoming translational barriers through advanced formulation and delivery strategies. As conjugation methods, nanodelivery technologies, radiotracing strategies, and AI-driven analytics continue to advance, phytochemicals may transition from early preclinical tools to clinically robust agents, either as standalone metabolic modulators or as critical components of combination strategies aimed at overcoming treatment resistance and improving patient outcomes.

## 7. Conclusions

Phytochemicals represent a promising and mechanistically diverse class of anticancer agents capable of targeting multiple metabolic vulnerabilities that sustain tumor growth, including the Warburg effect, the PPP, mitochondrial metabolism, and the TCA cycle. Evidence across diverse phytochemical classes, including polyphenols, terpenes, terpenoids, glucosinolates and alkaloids, demonstrates that these compounds interfere with distinct yet interconnected metabolic nodes by inhibiting glucose uptake, modulating key metabolic enzymes, disrupting mitochondrial function, and inducing redox imbalance. Importantly, individual phytochemicals may influence more than one metabolic pathway, highlighting a pattern of convergent and overlapping metabolic disruption rather than isolated pathway inhibition. Despite this therapeutic potential, challenges such as limited bioavailability, variability in phytochemical composition, and formulation complexity continue to hinder clinical translation. Advances in nanodelivery systems, drug conjugation strategies, and rational combination therapies offer promising solutions to overcome these barriers. Future studies should aim to systematically evaluate phytochemical-metabolic pathway interactions using standardized experimental frameworks and integrative metabolic profiling approaches. In this context, emerging tools such as metabolic imaging and real-time flux analysis may help identify pathway-specific vulnerabilities and guide personalized therapeutic strategies. By coupling broad metabolic targeting with precision delivery and translational validation, phytochemicals may emerge as versatile components of next-generation cancer therapies.

## Figures and Tables

**Table 1:** Examples of phytochemical classes, biological roles, and dosing considerations.

Phytochemical Class	Compound	In Vitro (Cell Line + Conc/IC_50_)	In Vivo (Animal Model + Dose/Regimen)	References
Polyphenols	Myricetin	In MDA-MB-231 breast cancer cells, myricetin (2.5, 5, 10, 20, and 40 μM) produced a dose-dependent suppression of cell viability	Xenograft mice with 4T1 cells; 20 or 50 mg/kg intravenous injection for 2 weeks. Tumor weight reduced.	[54]
Alkaloids	Berberine	IC_50_ values under hypoxia (dose range 0–20 µM): 4.14 µM (MDA-MB-231), 26.5 µM (MCF-7), 11.6 µM (4T1)	In the 4T1/Luc mouse model, oral berberine administration at 10–30 mg/kg body weight reduced tumor growth and altered the gut microbiota and metabolome	[55]
Isothiocyanate (glucosinolate-derived)	Sulforaphane (SFN)	SFN (5, 10, 20, 30, or 40 µM) showed dose-dependent cytotoxicity, with an IC_50_ of 19 µM in MCF-7 cells and 25 µM in SKBR-3 cells	Nude-mouse xenograft with MDA-MB-231 cells: daily injection 50 mg/kg for 2 weeks reduced cancer stem cell population in tumor	[56]
Terpenoid/Monoterpene	d-Limonene	d-Limonene (150, 300, 600, or 1200 µg/mL) showed dose-dependent cytotoxicity, with an IC_50_ of 246.05 µg/mL in A-375 cells and 2118.94 µg/mL in MDA-MB-468 cells	Breast: Rats (dietary) 2 g/day; Breast: Humans 2 g/day; Lung: Mice xenograft 400–600 mg/kg/day; Blood (Leukemia): Mice xenograft 0.5–1.5 mg/kg	[57,58]

**Table 2:** Metabolism-targeted radiolabeled probes for pet imaging in oncology.

Radiolabeled Agent	Target	Application	References
[^18^F] FDG (Fluorodeoxyglucose)	Glucose metabolism (Warburg)	Most common PET tracer for detecting various cancers	[123]
[^11^C] Methionine	Amino acid metabolism (protein synthesis)	Imaging of brain tumors (gliomas) and other tumors with high protein synthesis rates	[124]
[^18^F] Fluoroethyl tyrosine (FET)	Amino acid transport	Used in brain tumor imaging	[125,126]
[^11^C] Glutamine	Glutamine metabolism (glutaminolysis)	Studies tumors that rely on glutamine for energy and biosynthesis	[127]
[^11^C] Acetate	Lipid metabolism (fatty acid synthesis, energy production)	Used to study oxidative metabolism and lipid synthesis in cancers	[128]
[^18^F] Fluoromisonidazole (FMISO)	Hypoxia metabolism (oxygen deficiency in tumors)	Identifies hypoxic tumors, which are often therapy-resistant	[129]
[^11^C] Pyruvate	Lactate production in mitochondrial metabolism	Investigates metabolic changes in aggressive cancers and tumor energy production	[130]

**Table 3:** Clinical trials involving phytochemicals in cancer therapy and metabolism.

NCT Number		Title/Intervention Focus	Conditions	Location(s)
NCT01012141	Completed	Docetaxel with a Phytochemical in Hormone-Independent Metastatic Prostate Cancer	Metastatic Prostate Cancer	Clermont-Ferrand, France; Reims, France
NCT00433797	Completed	Dietary Intervention with Phytochemicals & Polyunsaturated Fatty Acids in Prostate Cancer	Prostate Cancer	Oslo, Norway
NCT06426771	Completed	Effect of AMH Levels on Inflammatory Index, Phytochemical Index & NRF Nutrient Density	AMH; Diet Habit; Ovarian Failure; PCOS	Ankara, Turkey
NCT03232138	Completed	Lung Cancer Chemoprevention with Sulforaphane in Former Smokers	Lung Cancer	Pittsburgh, PA, USA
NCT01961869	Active, not recruiting	Black Raspberry Confection in Oral Cancer Prevention	Healthy Volunteers	Columbus, OH, USA
NCT06600698	Recruiting	Safety & Efficacy of AGN-INM176 in Prostate Cancer Patients with Rising PSA	Prostate Cancer	Hershey, PA, USA
NCT01820299	Completed	Phase I Assay-Guided Trial of Anti-inflammatory Phytochemicals in Advanced Cancer	Solid Cancers	Charleston, SC, USA

## Data Availability

No datasets were generated or analyzed during the current study.

## Supplementary Materials

The following supporting information can be downloaded at: https://www.mdpi.com/article/10.3390/molecules31091394/s1, Supplementary Table S1. Classification of major phytochemical classes with representative polyphenols and their reported biological activities [133,134,135,136,137,138,139,140].
