# Supplementary material for: Plant-Derived Modulators of Tumor Metabolism as Novel, Efficacious, and Low-Toxicity Therapeutic Agents for Cancer Treatment
Source: Molecules. 2026 Apr 23;31(9):1394. doi: 10.3390/molecules31091394 (PMC13165046; doi:10.3390/molecules31091394)
Supplement: Supplementary file 1 [file molecules-31-01394-s001.zip › molecules-4232339-supplementary.pdf]

Supplementary Table S1. Classification of major phytochemical classes with representative polyphenols and their reported biological activities

Class	Representative Compounds	Biological Activities	Structure Examples	References
**Phenolic Acids**	Salicylic acid, Gallic acid, Caffeic acid, Ferulic acid	Antioxidant, anti-inflammatory, antimicrobial	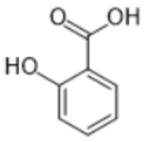 Salicylic acid 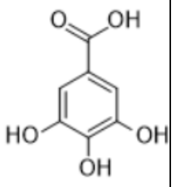 Gallic acid	[1], [2]
**Flavonoids**	Myricetin, Quercetin, Kaempferol, Luteolin	Antioxidant, anti-proliferative, immunomodulatory, antiangiogenic	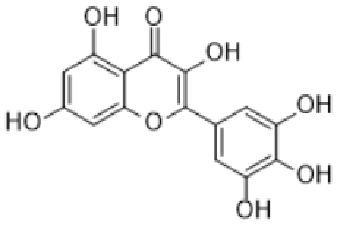 Myricetin	[3], [4]
**Tannins**	Ellagic acid, Catechins, Proanthocyanidins	Metal-chelating, antioxidant, anti-cancer	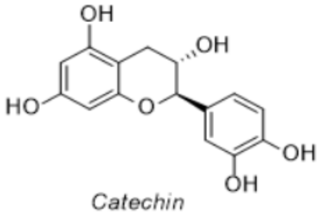 Catechin	[5], [6]
**Lignans**	Secoisolariciresinol, Matairesinol	Phytoestrogenic, antioxidant, hormone-modulating	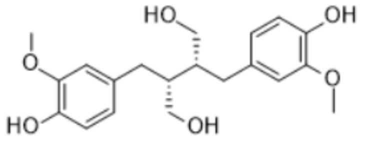 Secoisolariciresinol	[7]
**Coumarins**	Umbelliferone, Scopoletin	Anti-inflammatory, anti-proliferative, antioxidant, antimicrobial	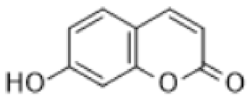 Umbelliferone	[8]

References

1. Tsepaeva OV, Salikhova TI, Grigor'eva LR, Ponomaryov DV, Dang T, Ishkaeva RA, et al. Synthesis and in vitro evaluation of triphenylphosphonium derivatives of acetylsalicylic and salicylic acids: structure-dependent interactions with cancer cells, bacteria, and mitochondria. Medicinal Chemistry Research. 2021;30(4):925-39.10.1007/s00044-020-02674-6
2. Valanciene E, Jonuskiene I, Syrpas M, Augustiniene E, Matulis P, Simonavicius A, et al. Advances and Prospects of Phenolic Acids Production, Biorefinery and Analysis. Biomolecules. 2020;10(6).10.3390/biom10060874
3. Paravati MR, Scarlata GGM, Milanović M, Milić N, Abenavoli L. The anticancer activity of quercetin, luteolin, myricetin, and kaempferol in the development of hepatocellular carcinoma: a narrative review. Hepatoma Research. 2024;10:N/A-N/A,

4. Felice MR, Maugeri A, De Sarro G, Navarra M, Barreca D. Molecular Pathways Involved in the Anti-Cancer Activity of Flavonols: A Focus on Myricetin and Kaempferol. International Journal of Molecular Sciences. 2022;23(8):4411, https://www.mdpi.com/1422-0067/23/8/4411
5. Farhan M. Green Tea Catechins: Nature's Way of Preventing and Treating Cancer. International Journal of Molecular Sciences. 2022;23(18):10713, https://www.mdpi.com/1422-0067/23/18/10713
6. Moreno JAJ, Ferreira VC, Ampese LC, de Freitas Marinho L, Rostagno MA, Carneiro TF. An overview of the ellagic acid and proanthocyanidins' polyphenols from cambuci (Campomanesia Phaea Berg): Myrtaceae's family. European Food Research and Technology. 2024;250(3):859-76.10.1007/s00217-023-04413-8
7. Andargie M, Vinas M, Rathgeb A, Möller E, Karlovsky P. Lignans of Sesame (Sesamum indicum L.): A Comprehensive Review. Molecules. 2021;26(4):883, https://www.mdpi.com/1420-3049/26/4/883
8. Kornicka A, Balewski Ł, Lahutta M, Kokoszka J. Umbelliferone and Its Synthetic Derivatives as Suitable Molecules for the Development of Agents with Biological Activities: A Review of Their Pharmacological and Therapeutic Potential. Pharmaceuticals. 2023;16(12):1732, https://www.mdpi.com/1424-8247/16/12/1732
